# Designing with Iontronic Logic Gates—From a Single Polyelectrolyte Diode to an Integrated Ionic Circuit

**DOI:** 10.1021/acsami.3c00062

**Published:** 2023-04-17

**Authors:** Barak Sabbagh, Noa Edri Fraiman, Alex Fish, Gilad Yossifon

**Affiliations:** ‡ Faculty of Mechanical Engineering, Technion−Israel Institute of Technology, Haifa 3200003, Israel; § Faculty of Engineering, Bar-Ilan University, Ramat Gan 5290002, Israel; ⊥ School of Mechanical Engineering, Tel Aviv University, Tel Aviv 69978, Israel

**Keywords:** iontronic, ionic diode, polyelectrolyte, ionic logic gate, ionic circuitry

## Abstract

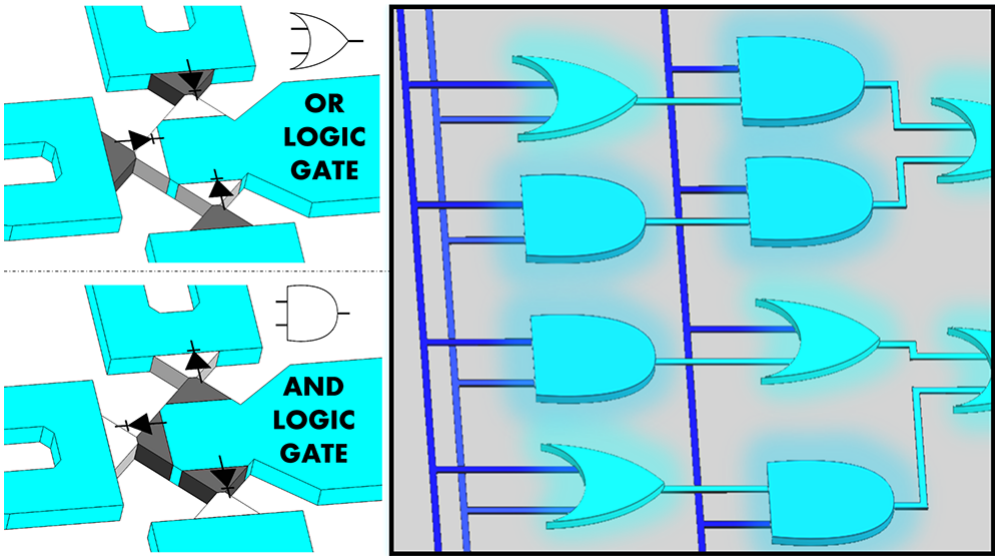

This article presents the implementation of on-chip iontronic circuits via small-scale integration of multiple ionic logic gates made of bipolar polyelectrolyte diodes. These ionic circuits are analogous to solid-state electronic circuits, with ions as the charge carriers instead of electrons/holes. We experimentally characterize the responses of a single fluidic diode made of a junction of oppositely charged polyelectrolytes (i.e., anion and cation exchange membranes), with a similar underlying mechanism as a solid-state p- and n-type junction. This served to carry out predesigned logical computations in various architectures by integrating multiple diode-based logic gates, where the electrical signal between the integrated gates was transmitted entirely through ions. The findings shed light on the limitations affecting the number of logic gates that can be integrated, the degradation of the electrical signal, their transient response, and the design rules that can improve the performance of iontronic circuits.

## Introduction

Biological cell membranes contain multiple proteins that act as channels and enable a highly selective exchange of ions and molecules. These channels interact with each other and allow for complex signaling circuits that regulate the transmembrane potential.^1^ As compared to artificial circuits, the membrane’s threshold response is akin to that of a digital system. Artificial digital logic gates based on ionic signal transmission can thus mimic biological systems and also function as an electrical circuit. They constitute the emerging research field known as iontronics.^2−5^ Iontronic devices usually contain nanometer-sized fluidic structures (e.g., nanochannels, ion exchange membranes) that exhibit ion permselectivity due to the overlap of their electric double layers (EDLs).^6^ Their unique electrical behavior enables them to perform in vitro/vivo information processing at the ionic level using iontronic components such as resistors, diodes, capacitors, and transistors.^4,7,8^ These nanofluidic components are attracting intensive study, given their fundamental interest and promising applications beyond biomimetic information processing that can contribute to improving chemical and biochemical sensing,^9,10^ such as for energy harvesting,^11^ single molecule detection,^12^ electrokinetic preconcentration,^13,14^ and brain–machine interfaces.^15^ Some of the best-known electrical gates for Boolean logic computation consist of diodes, i.e., diode-based logic gates (DLGs). Diodes are two-terminal components exhibiting a nonlinear current–voltage response (I–*V*) that have a higher conductance in one current flow direction (forward-biasing) than in the reverse direction (reverse-biasing). They can be characterized by their rectification ratio, R, which is defined as the rate between the forward- (I_F_) and the reverse-biased (I_R_) currents for opposite voltage polarities (R = |I_F_/I_R_|). Regulation of the electrical current (I) by applying a voltage (*V*) is critical for the implementation of a logic gate. In DLG, the higher the R, the more precise is the control over the current and the higher is the gate’s performance. Integrating resistors within the circuit provides the constant load resistance needed for the functionality of the gates.

In solid-state electronics, the circuit architecture connecting the diodes and resistors determines the type of DLG. Two types of Boolean functions can be realized by DLG: OR and AND. Each receives a number of logic inputs and returns a single logic output of disjunction/conjunction (for OR/AND DLG, respectively). Specific input and output potential levels are assigned to either “high” or “low” binary logic levels that are labeled “1” or “0”, respectively. Previous studies have shown that basic ionic circuitry up to a level of sophistication of an individual DLG can be realized with the same principles as solid-state electronics by using either unipolar or bipolar nanofluidic diodes immersed in an electrolyte solution (e.g., aqueous KCl).^16−26^ Unipolar nanofluidic diodes have been made using a geometric symmetry-broken fabricated conical nanopore^27−29^ or a funnel-shaped nanochannel^30,31^ that slightly favors the ionic current in one direction. However, the maximum R of these diodes rarely exceeds one order of magnitude, which makes it difficult to create an efficient DLG. Improvement of the rectification ratio can be achieved by filling the nanostructure with a nanoporous membrane made of a polyelectrolyte.^32^ A more practical realization with a higher R consists of a bipolar diode made of a junction of two oppositely charged ion permselective regions. Surface-functionalized nanochannels,^33^ field-effect nanochannels,^34^ nanoparticles,^35^ and anionic- and cationic exchange membranes (AEM and CEM, respectively) such as polyelectrolytes^16,17,22,36−38^ have been used to that end. Under reverse bias, both mobile cations and anions (positively and negatively charged ions, respectively) are depleted from the junction, which results in significantly decreased conductance. Reversing the direction of the electric field to forward bias transitions the ionic depletion into an ionic enrichment at the junction, which recovers to increased conductance.

The mechanism underlying the nanofluidic bipolar diode is similar to an electronic solid-state p- and n-type (p-n) junction diode that uses electrons and holes instead of ions as the free charge carriers. Nevertheless, there are fundamental differences between fluidic and solid-state devices in that ion transport is much more complicated. Its complexity stems from electrochemical electron–ion exchanges, the significantly lower mobility of ions compared to electrons, the variety of ionic species, the lack of ionic charge recombination, and fluid flow effects.^5,39^ On the one hand, based on these unique properties, a rich variety of applications can be realized using ionic diodes, e.g., separation, gating, and sensing of ions or even power generation.^40−43^ On the other hand, all of the mentioned differences are expected to have a major impact on the realization of a DLG using these diodes, and in particular on the integration of several DLGs into multistage circuits, which can further degrade its performance due to current leakages and parasitic resistances. To the best of our knowledge, the well-known behavior of solid-state DLG-based integrated circuits^44^ has not been investigated in iontronic DLG-based fluidic circuits. Apart from the increased complexity involved in the physical description of ion transport relative to that of electrons, the challenge of a robust and reliable fabrication and integration of multiple ionic diodes onto a single chip has further made it difficult for implementation.

Here, we report the small-scale integration of a bipolar polyelectrolyte diode-based iontronic circuit within a microfluidic chip. Whereas the mechanism underlying the operation of a single diode has been studied extensively, we focused on its ability to construct a DLG. The fluidic system, including the diodes and interconnecting microchannels, was built on the basis of a double-sided adhesive sheet that was patterned (i.e., cut) according to a predefined circuit architecture along with photocuring of the polyelectrolytes to form bipolar junctions. This relatively simple but reliable technique enabled us to design and develop in-plane circuits without any limitation on the number of the diodes, their locations, and their orientation. The experimentally realized iontronic DLGs were then examined in terms of switching speed, voltage shifting, noise margin, and cascading capabilities, all inspired by the world of solid-state electronics. These enabled us to realize more complex iontronic logic functions with varying circuits by integrating multiple DLGs, where the electrical signal between the integrated DLGs is transmitted entirely through ions.

## Results and Discussion

### Characteristics of a Single Bipolar Nanofluidic Diode

**Figure 1:**
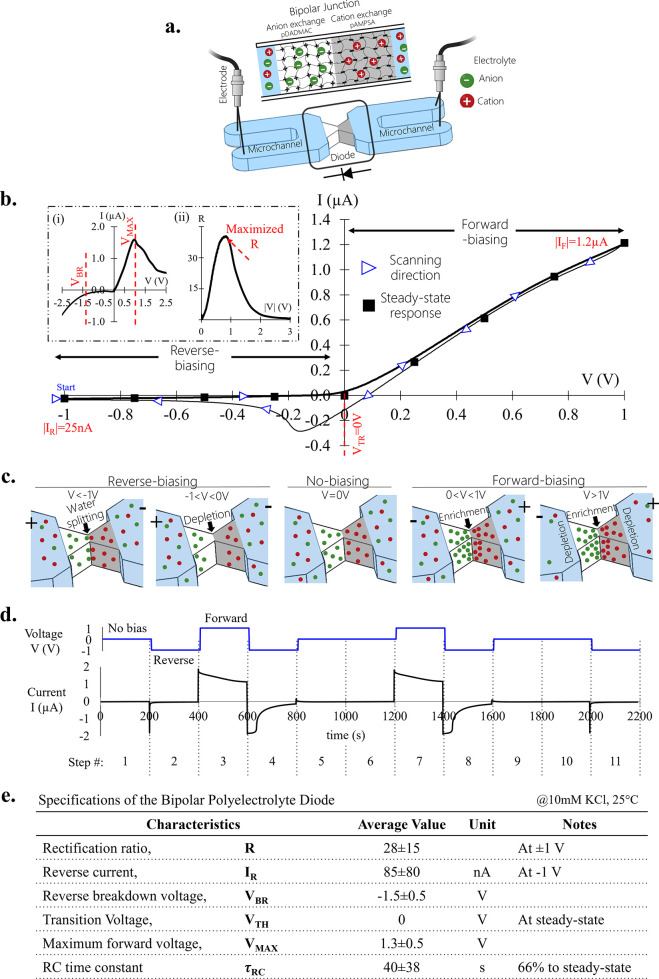
Characterization of a single fluidic bipolar diode’s response. (a) Schematics of a bipolar fluidic diode with a cationic–anionic membrane-based junction. The arrow points in the direction of the high conductance current flow of the diode. (b) Current–voltage (I–*V*) response to a voltage scan from −1 V to +1 V and back to −1 V at a scan rate of 100 μV/s. The arrow heads indicate the scanning direction. Solid black squares indicate the steady-state response of the amperometric measurements under a constant applied *V* after 500 s. Inset: (i) I–*V* response for −2.5 < V< +2.5 V; (ii) calculation of the rectification ratio, R, for the *V* taken from (i), where the maximum R was obtained at ±1 V. (c) Representative electrolyte ion distribution inside and outside the diode’s junction for various *V* values. (d) Experimental current I response over time (bottom graph, black line) for various voltage *V* steps (top graph, blue line). Steps 1, 5, 6, 9, 10 no-bias *V* = 0 V; steps 2, 4, 8, 11 reverse bias *V* = −1 V; steps 3, 7 forward bias *V* = +1 V. (e) Specifications of the bipolar polyelectrolyte diode. The electrical characterization, including the mean value and its standard deviation, was based on 15 diodes.

A single bipolar polyelectrolyte diode interconnected to two microchannels was electrically characterized for 10 mM KCl [Figure 1] (see also Methods section and supplementary Figures S1−S3). At steady-state operation, the transition voltage (*V*_TR_) closing the open diode occurred at *V*_TR_ ≈ 0 V, where the ion concentrations at the junction are controlled by the Donnan equilibrium.^45^ At positive voltages (0 < *V* < +1 V), where the diode is considered open, the forward-biased current increased linearly with increasing voltage and reached a value of |I_F_| ≈ 1200 nA at *V* = +1 V [Figure 1b]. At negative voltages (−1 < *V* < 0 V), where the diode is considered closed, the reverse-biased current was significantly smaller and almost totally independent of the applied voltage, reaching a value of |I_R_| ≈ 25 nA at *V* = −1 V. The nonideal permselectivity of the ion exchange membranes prevented I_R_ from dropping to zero current under reverse bias. This nonideality became worse with higher electrolyte ionic strength, resulting in an enlarged I_R_ [Figure S4]. Geometric flaws in the fabricated polyelectrolyte membranes also significantly enlarged the I_R_ as a result of ion transport that bypassed the membranes [Figure S5]. The transient I–*V* scan (100 μV/s) from reverse- to forward- and back to reverse bias (−1 V → +1 V → −1 V) revealed a hysteresis of the current response around 0 V due to residual ion enrichment at the junction. This residual concentration of ions stems from their slow diffusive transport relative to the temporal changes of the voltage, which resulted in an apparent negative differential resistance.^5,21,37,38^ The hysteresis grew with increased scanning rate [Figure S6] while shifting to negative voltages the transition point at which the diode was closed, and completely vanished in the steady-state response [Figure 1b]. This contrasts with solid-state diodes that exhibit a constant potential barrier (a typical value of *V*_TR_ ≈ +0.7 V) to open the diode in high operation frequencies and without a hysteresis effect due to the significantly larger electron mobility and possible recombination of electrons and holes at the junction.^5,44^ Increasing the voltage range beyond |1 V| introduced additional effects that further complicated the ionic diode’s response. Under reverse-biasing, water splitting into H+ and OH– generated excess mobile charge carriers that further elevated the I_R_ once the electric field at the junction exceeded an order of MV/cm.^46,47^ Based on the obtained current response, we evaluated the ability to reach these values as crossing a reverse breakdown voltage of *V*_BR_ ≈ −1.4 V. In addition, under forward-biasing, generation of ionic depletion regions at the microchannel–polyelectrolyte interfaces due to ion-concentration polarization (ICP) was present as well, resulting in reduced conductance.^39^ This led to the appearance of a maximum I_F_ at a given forward voltage, *V*_MAX_ ≈ 1.2. Both water splitting and ICP effects resulted in a narrow voltage range of ∼|1 V| where the rectification ratio reached a maximum value of R = 48 at ±1 V. Stepwise chronoamperometry (stepping *V* and monitoring I as a function of time, Figure 1d) was used to measure the current’s temporal response at different applied voltages at a switching speed of 5 mHz. Each step in the potential (steps 1, 5, 6, 9, 10 *V* = 0 V; steps 2, 4, 8, 11 *V* = −1 V; steps 3, 7 *V* = +1 V) simulated a different activation mode that the diode would experience when used to realize a DLG. The findings showed that the current I reached a repeatable equilibrium value for each mode, regardless of the preceding step that could affect its transient response and RC time constant (defined as the time needed for an RC circuit to reach 63% of its steady-state value). Comparing the RC time obtained for the diode (O(10^1^ s)) to that of a typical solid-state diode (O(10^–9^ s))) clearly underscored the difference between ionic mobilities, which were several orders of magnitude smaller than the mobilities of electrons/holes.^2^ The diode’s characteristics that we obtained are summarized in Figure 1e. When compared to solid-state diode characteristics (depicted in Table S1), it is clear that these emerged as fundamentally different. This highlights the need to further investigate the ability of bipolar polyelectrolyte diodes to realize iontronic DLG as well as integrated circuits made of several DLGs.

Besides the bipolar junction, the interconnecting microchannels have a major impact on the overall response. Ideally, these microchannels should have a negligible effect; however, they exhibited ∼80% of the system’s total voltage drop under forward bias and ∼4% under reverse bias (at ±1 V) [Figure S7]. This microchannel-related parasite resistance continued to rise with an increasing number of diodes and their associated number of interconnecting microchannels. For lower ionic strength (below ∼1 mM), the role of the interconnecting microchannels on the overall resistance was expected to increase.^48^ Thereby, a 10 mM KCl electrolyte was chosen as the most suitable working solution that met the requirements of yielding sufficiently large R without a too large microchannel resistance.

### Characteristics of an Individual Iontronic DLG (OR/AND)

**Figure 2:**
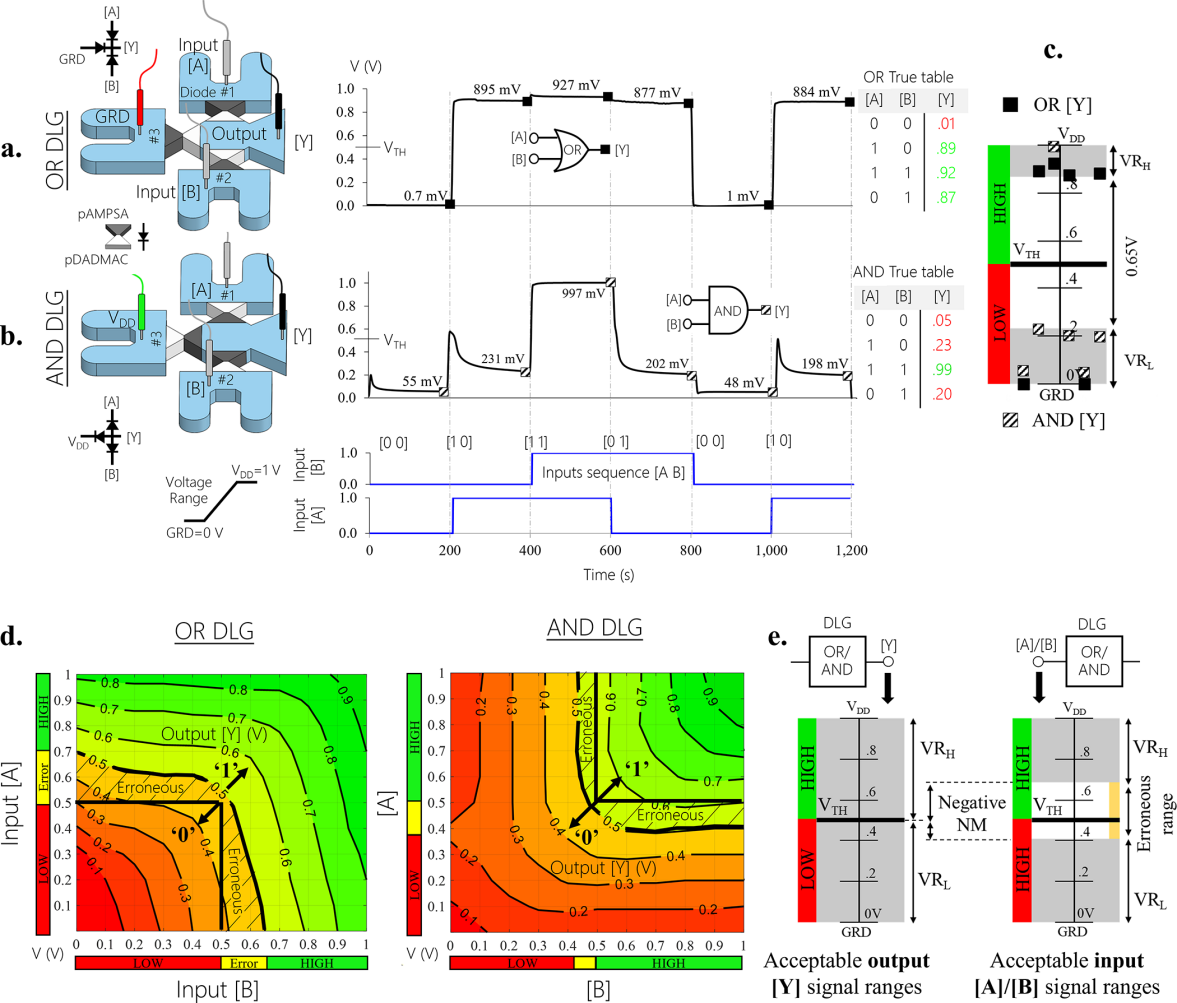
Individual DLG (diode-based logic gate) response (OR/AND). (a) OR DLG. (b) AND DLG. Green and red represent high (1) and low (0) logic levels, whereas a threshold voltage of +0.5 V (=*V*_TH_) separates the two. From left to right: Schematics of the fluidic system, containing three bipolar polyelectrolyte diodes, interconnecting microchannels, two voltage inputs [A] and [B] that receive either 0 V (=GRD) or +1 V (=*V*_DD_), and a measured output voltage [Y]. Measured [Y] over time (black line) for various logic input sequences (blue line). Truth table (in volts). (c) Plot of the output readouts of both DLGs together, where deviations from *V*_DD_ and GRD (marked in gray) were considered a high voltage range (*V*R_H_) and a low voltage range (*V*R_L_), respectively. (d) Response diagrams of the DLGs for varying voltage inputs (0–1 V) showing the voltage range of inputs (0.5−0.7 V for OR and 0.4−0.5 V for AND DLG) that led to an error in the logical interpretation of [Y] (defined as the erroneous range, marked in yellow). (e) Noise margin (NM) plot of the acceptable output signal ranges versus input signal ranges for both AND and OR DLGs.

Based on the diode’s characteristics, we designed and experimentally examined iontronic DLGs [Figure 2]. In our ionic circuits, low (0) and high (1) logical inputs were realized by directly applying 0 V (=GND) and +1 V (=*V*_DD_), respectively. A threshold voltage of +0.5 V (=*V*_TH_)was defined to distinguish between the two output logic levels. An output potential reading ([Y]) below *V*_TH_ was defined as a low logical level (0) and above as a high level (1). All our circuits were similarly assembled from a symmetric arrangement of three diodes connected by interconnecting microchannels to achieve better performance, where the direction of the diodes dictated the DLGs’ functionality. OR DLG was implemented by interfacing the conductive direction of the diodes inward to the circuit’s center versus outward for AND DLG. Two parallel inputs ([A B]) were introduced into the side diodes (diodes #1, 2), while the central diode (#3) was constantly kept closed to act as a load resistance biased to GRD/*V*_DD_ (for OR/AND DLG, respectively). [Y] was obtained at the center of the circuit between the three diodes. For OR DLG [Figure 2a], if at least one of the inputs was high, the corresponding side diodes became forward-biased. Thus, the current, *I*, passed freely across those diodes with a minimal voltage drop, yielding a high potential at the output measuring point [Y]. [Y] was experimentally measured for various input sequences (a total of 2^2^ possible input sequences), and the stabilized signal after 200 s for each input sequence was summarized in a truth table. The long stabilization time stemmed from that of a single ionic bipolar diode. As expected, low [Y] with a measured voltage approaching GRD (1 mV ≪ *V*_TH_) was only obtained when both inputs were low ([A B] = [0 0]). High [Y] was obtained for all other sequences with an average [Y] of 900 mV (>*V*_TH_) with a minimum readout of 877 mV ([0 1]). Hence, in the transition the applied *V*_DD_ was degraded across parasite resistances including the forward-biased diodes and microchannels, whereas leakage current through the reverse-biased diodes further enhanced the degradation to ∼10% of *V*_DD_. As the entire iontronic circuit shared the same electrolyte solution using interconnecting microchannels, reducing the parasite resistances was obtained through geometrical modifications (e.g., increased width and decreased length) of the microchannels. The small [Y] variations between opposite inputs (e.g., [1 0]:895 mV and [0 1]:877 mV) were likely due to physical differences between the diodes (see Figure S8 indicating the variation in R obtained for the different diodes). For AND DLG [Figure 2b], most of the voltage drop occurred across the reverse-biased side diodes that received high input. The resulting truth table showed output [Y] characterized as a low logic level with voltage variations below *V*_TH_ between ∼50 ([0 0]) and ∼230 mV ([1 0]) depending on which and how many inputs were set to low. [Y] only became high with a potential approaching *V*_DD_ (997 mV ≫ *V*_TH_) when both inputs were set to high ([1 1]). Examining the transient responses of both OR and AND DLGs revealed similar RC time constants as that of a single diode (O(10^1^ s)) due to the gate’s parallel inputs. Repeating the same input sequence ([1 0]) at the beginning and end of the operation confirmed the repeatability of the DLGs. Plotting all of the final output readouts from the individual DLGs revealed the deviations of [Y] from an ideal gate response [Figure 2c]. A deviation from *V*_DD_ was considered a high voltage range (*V*R_H_), and a deviation from GRD was considered a low voltage range (*V*R_L_). The smaller the parasite resistances, the lower *V*R_H_ and *V*R_L_ would be. In spite of the sufficiently large voltage gap between *V*R_H_ and *V*R_L_ (∼0.65 V), indicating that the output voltage had attained the correct logical level, the signal deviations limited the number of DLGs that could be cascaded. Investigating the output readouts for a range of input voltages from low to high (0–1 V) identified [Figure 2d] the dependency of the output on the input signals. High input below 0.7 V (but above *V*_TH_) to OR DLG could degrade [Y] below *V*_TH_ and result in a faulty logic interpretation of a low level. Similarly, a low input above 0.4 V (but below *V*_TH_) to AND DLG did not always provide a correct logic level of low [Y]. Hence, this erroneous range (0.4–0.7 V) should be avoided as an input to ensure proper logical functionality of the cascaded DLG. Nevertheless, in cascading DLGs in a series (when each DLG in the series is considered a stage), the output of the preceding DLG drove the input of the subsequent DLG. The fact that the DLGs’ acceptable output voltages (0–1 V) overlapped with this erroneous voltage range would ultimately negate the ability of the subsequent DLG to function as required (defined as a negative noise margin) [Figure 2e].

### Logical Computation by Integration of Multiple Iontronic DLGs

**Figure 3:**
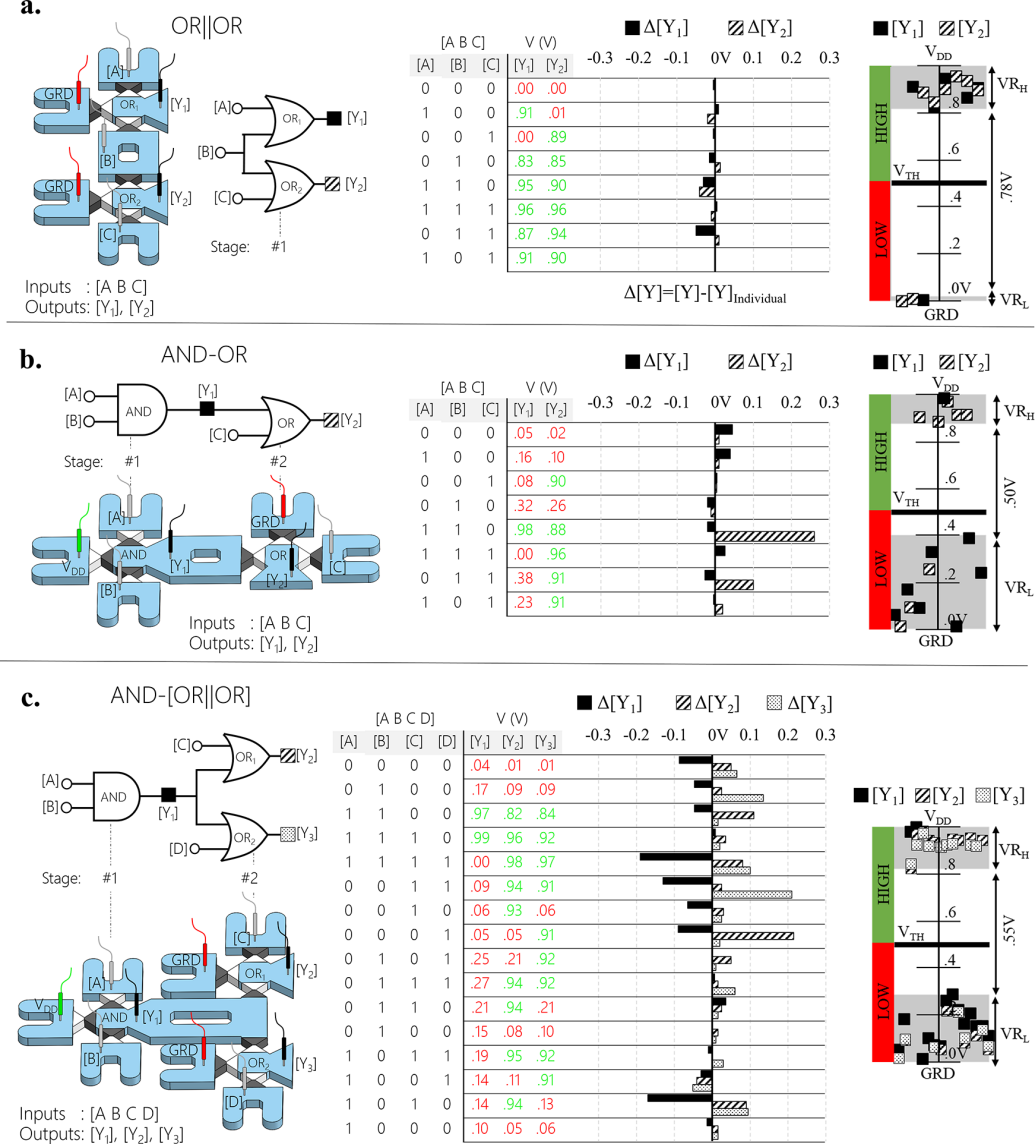
Integration of multiple diode logic gates (DLGs). (a) Two OR DLGs connected in parallel with a common input [B] (OR||OR). (b) AND DLG connected in series with OR DLG (AND-OR) where the AND DLG’s output [Y_1_] served as the input of the OR DLG. (c) Circuit of AND DLG connected in series with two OR DLGs that were connected in parallel (AND-(OR||OR)). (a–c) Left to right: Schematic of the fluidic system and logic circuit diagram, truth table, output voltage deviations for each input sequence shown in the truth table, and a plot of the output readouts. Red and green represent low (“0”) and high (“1”) logic levels, respectively. Δ[Y] = ([Y]−[Y]_Individual_) is defined as the difference between the output of the integrated ([Y]) and the non-integrated gate ([Y]_Individual_). [Y]_Individual_ was obtained by deactivating all other DLGs.

New logic functions with varying numbers of DLGs, stages, inputs, and outputs were realized by integrating multiple DLGs. Each DLG was operated as part of an integrated DLG circuit and as an individual DLG (nonintegrated by deactivating all other DLGs), allowing us to inspect the effect of integration on the outputs by comparing the output values (Δ[Y] = [Y] – [Y]_Individual_). Initially, we examined basic integration configurations such as two DLGs connected in parallel (OR||OR) and in series (AND-OR) [Figure 3]. The parallel circuit was designed to consist of a single stage of two OR DLGs with a total of three inputs ([A B C]) and two outputs ([Y_1_], [Y_2_]) [Figure 3a]. A common input ([B]) connected both DLGs by using a common interconnecting microchannel. An effect of the integration of up to +50 mV increase of VR_L_ of both outputs was obtained as inputs ([A C]) drove through the common interconnecting microchannel not only their own DLG. In the series circuit, the output of the first stage (AND with [Y_1_]) was connected to one of the two inputs of the second stage (OR with [Y_2_]) [Figure 3b]. Such an integration resulted in a significant signal deviation of [Y_2_] expressed in increased VR_L_ by 0.25 V. Furthermore, minor variations of [Y_1_] (±50 mV), obtained due to activations of input [C], were associated with the input of the second stage. Hence, not only does the previous stage affect the following one (as was predicted based on the individual DLGs’ responses) but also vice versa. Nevertheless, the results showed that the DLGs could be integrated in both ways while maintaining the correct output logic level for all 2^3^ possible input sequences. None of the output readings fell within the erroneous logical output’s voltage range, suggesting that it could be further integrated with additional DLGs. We then successfully realized a more complex circuit that combined integration in series and parallel composed of a two-stage cascade with three DLGs that included an AND DLG that drove two parallelly connected OR DLGs (AND-[OR||OR]) [Figure 3c]. Although a single output ([Y_1_]) drove two inputs, the deviations remained the same and did not cause a logical failure.

**Figure 4:**
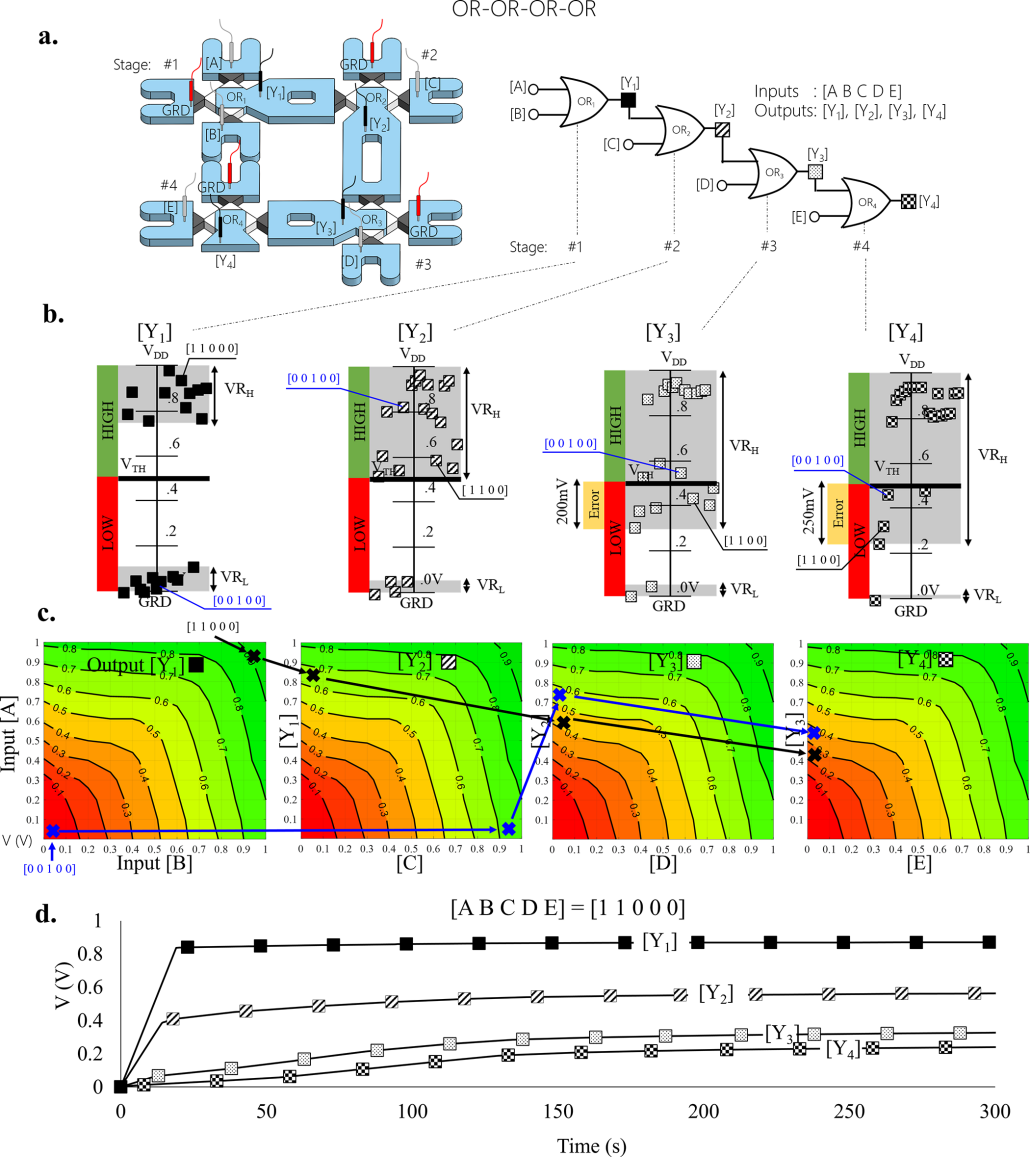
Integration of four OR gates connected in series. (a) Gate schematic and its fluidic implementation. (b) Plots of the readouts for the output of each stage ([Y_1_], [Y_2_], [Y_3_], [Y_4_]). Green and red represent logic levels 1 and 0, and yellow indicates a faulty readout. The full truth table can be found in Table S2. (c) Prediction of the circuit behavior based on individual DLG responses (response diagrams taken from Figure 2). Two input sequences (of inputs [A B C D E]) were examined: [1 1 0 0 0] (black line) and [0 0 1 0 0] (blue line). X indicates the predicted output voltages, and the arrows indicate the signal propagation path. (d) DLG responses over 300 s for high level (=*V*_DD_) inputs [A] and [B]. The remainder were low level (=GRD) ([1 1 0 0 0]).

The integration limit was reached by cascading four OR DLGs in series (OR-OR-OR-OR, total 4 stages) [Figure 4]. Probing the outputs revealed a monotonic signal degradation at every stage that eventually resulted in a false logic readout [Figure 4a]. Out of 21 examined input sequences, 8 yielded false logic (marked in yellow); for example, degradation from ∼0.9 V (>*V*_TH_) at the first stage (high [Y_1_]) to ∼0.3 V (<*V*_TH_) at the fourth stage (low [Y_4_]), although all DLGs should have exhibited a high logic level for the input sequence ([A B C D E] = [1 1 0 0 0]). The false readouts started from the third stage since the second stage’s output fell within the erroneous logical output’s voltage range (0.4 < [Y_2_] = 0.6 < 0.7 V). Thus, the behavior of the cascade was predicted based on the individual DLG’s response [Figure 4b]. Inspecting the same input sequence ([A B C D E] = [1 1 0 0 0]) in the response diagrams, where each diagram represents a stage, revealed a matching trend. We thus used this inspection technique for feasibility studies of other logic functions without having to realize them. For example, a cascade consisting of two OR and AND DLGs connected in series (OR-OR-AND, total of three stages) showed reliable output results [Figure S9]. For verification, this circuit was experimentally realized and exhibited correct logical readouts as expected. A successful cascading of pairs of AND and OR DLGs with a total number of 5 DLGs integrated in series (AND-OR-AND-OR-AND) was shown to work theoretically and to exceed the limit of a circuit consisting more than 3 working stages. Potentially this circuit could be extended to include additional DLGs [Figure S10]. Along with the voltage outputs, the RC time constant was also considered. It was proportional to the number of DLGs through which the ions were transported [Figure 4C]. Although the first stage acquired a saturated signal within tens of seconds (O(10^1^ s)), the fourth stage only reached saturation after a few hundred seconds O(10^2^ s) due to its dependency on the ionic signal of the previous DLGs.

## Conclusions

We described the implementation of on-chip iontronic circuits using a small-scale integration of diode-based logic gates (OR/AND gate) consisting of bipolar polyelectrolyte diodes. The single diode characteristics (e.g., rectification ratio, operation voltages, and RC time constant) determined the operating conditions of the fluidic diode-based logic gates. Although the mechanisms underlying the operation of a bipolar polyelectrolyte diode and a p-n solid-state diode are similar, their characteristics were found to be fundamentally different, mainly due to the significantly lower mobility of ions relative to that of electrons, multiple ionic species, as well as complicating diffusive and convective effects. After taking all of the circuit components (i.e., diodes, interconnecting microchannels, and electrolyte’s ionic concentration) in the microchip architecture into account, fluidic diode logic gates that exhibited a consistent and robust differentiation between the logic levels were successfully obtained. We then successfully integrated several such diode logic gates into different circuit architectures, demonstrating for the first time that a real ion-permselective membrane based iontronic integrated circuit can be designed to perform in-chip computation on various inputs. However, we found a limitation on the number of logic gates that could be successfully integrated, which stemmed from the drift of the output voltage with each subsequent gate due to undesired leakage currents and parasite resistances. Eventually, when the output drift was large enough, it resulted in a faulty logic readout. Thus, simulation tools that take all of the integration effects into account, in addition to the responses of a single logic gate, are crucial to the design of an integrated ionic circuit consisting of many logic gates, similar to what is implemented in the design of very-large-scale (VLSI) electronics circuits. Implementing the gained understanding and design rules for practical iontronic devices can improve their performance and enable more advanced and complex ionic computing functions as more ionic processing units will be integrated. However, to fully realize the potential for more complex logic functions with a larger number and types of logic gates would require including both fluidic transistors and diodes in constructing the logic gates to enable amplification of the signal. This type of amplification is essential for signal regeneration throughout the circuit and can only be achieved by integrating fluidic transistors as well.

## Methods

### Materials

3 M Optically Clear Adhesive 8146-1-ND, 3-(trimethoxysilyl)propyl methacrylate, 4.2 M diallyldimethylammonium chloride (DADMAC), 4.2 M 2-acrylamido-2-methyl-1-propanesulfonic acid (AMPSA), 2-hydroxy-4′-(2-hydroxyethoxy)-2-methylpropiophenone, *N*,*N*′-methylenebis(acrylamide), H_2_SO_4_(%)/H_2_O_2_(%) = 3:1.

### Fabrication of the Microfluidic Chip with Integrated Polyelectrolyte Diodes

Thin double-sided adhesive tape (∼25 μm in width) was sandwiched between two glass slides (70 × 50 mm, Sigma) and used for patterning the microfluidic channels by cutting the tape (Silhouette Cameo 4). The upper slide was then drilled with holes (1.8 mm diameter) as inlets for the microchannels. The polyelectrolyte bipolar diodes were situated within the microchannels at designated narrow locations (300 μm junction minimum width) by polymerization of diallyldimethylammonium chloride (DADMAC) and 2-acrylamido-2-methyl-1-propanesulfonic acid (AMPSA) face to face through UV-light exposure. Both poly DADMAC and AMPSA exhibit high ion permselectivity, electrochemical stability, and water solubility.^49,50^ Furthermore, both were widely used in previous studies related to microfluidic applications,^51,52^ and in particular in realizing bipolar diodes.^37,38,47^ Their photoresponsive cross-linking capability and strong adhesion to silica-based substrates (i.e., glass slides) make them ideal for use within microfluidic devices. Additional information on the fabrication process is provided in Figure S1.

#### Electrical Measurements

Silver–silver chloride electrodes (Ag/AgCl, A-M Systems, 0.015 in. diameter) were used for the electrical measurements including current–voltage (I–*V*, scan rate of 100 μV/s), chronoamperometry, DLG activation by biasing either 0 V or +1 V to each DLG’s input, and open-circuit potential measurements (zero net current, I = 0) to obtain the DLG’s output voltage. A 12-channel automatic relay system (custom-made) was used to expand controllability over two potentiostats (Keithley 2636, Gamry Reference 3000) toward multiple DLGs. Additional information on the fabrication process and the data acquiring method is provided in Figures S2 and S3.

## Supporting Information Available

Fabrication of the microfluidic chip with integrated polyelectrolyte diodes, acquisition of the circuit’s output readouts, experimental setup, effect of the electrolyte ionic strength on the bipolar diode performance, variations in the bipolar polyelectrolyte diode fabrication and its influence on the rectification factor, current–voltage response of a single bipolar diode at two different scan rates, estimation of the forward- and reverse-biased diode resistances, rectification ratios of the used bipolar polyelectrolyte diodes, successful integration of three diode logic gates in series, theoretical output prediction of the integration of five diode logic gates connected in series, typical values of solid-state p-n electronic diode, and truth table of the circuit in Figure 4 (PDF)
