# Supplementary material for: Designing with Iontronic Logic Gates—From a Single Polyelectrolyte Diode to an Integrated Ionic Circuit
Source: ACS Appl Mater Interfaces. 2023 Apr 17;15(19):23361–70. doi: 10.1021/acsami.3c00062 (PMC10197067; doi:10.1021/acsami.3c00062)
Supplement: Supplementary file 1 — am3c00062_si_001.pdf [file am3c00062_si_001.pdf]

Supporting Information

Designing with Iontronic Logic Gates - From a Single Polyelectrolyte Diode to an Integrated Ionic Circuit

Barak Sabbagh[1], Noa Edri Fraiman[2], Alex Fish[2*], Gilad Yossifon[1,3*]

[1]*Faculty of Mechanical Engineering, Technion–Israel Institute of Technology, Haifa, Israel*

[2] *Faculty of Engineering, Bar-Ilan University, Ramat Gan, Israel*

[3]*School of Mechanical Engineering, Tel-Aviv University, Tel Aviv, Israel*

Corresponding authors: alexander.fish@gmail.com, gyossifon@tauex.tau.ac.il

Fabrication of the microfluidic chip with integrated polyelectrolyte diodes

Step 1 – patterning the double-sided adhesive. The first step was a computer-aided design (CAD) drawing of the chip's architecture, including the interconnecting channels, inlets, outlets, and designated locations for the diodes. The interconnecting microchannels were designed such that the solution within could be easily exchanged. Unlike standard diode-based logic gates consisting of two diodes and a resistor, all our circuits were similarly assembled from a symmetric arrangement of three diodes connected by interconnecting microchannels to achieve better performance, where the direction of the diodes dictated the DLGs' functionality. The symmetric configuration of the diode-based logic gate minimized these variations by using interconnecting microchannels of the same resistance for any input. Next, the design was imported to a blade-based automatic cutting machine (CAMEO silhouette 4), where a thin double-sided adhesive covered with protective sheeting on both sides was cut according to the outer lines of the CAD drawing. The double-sided adhesive (3M™ Optically Clear Adhesive 8146-1-ND) exhibited excellent resistance to aqueous solutions, at a low thickness of 25µm, that is low in cost, and most importantly resulted in well-defined edges and minimal distortion with a lateral cutting resolution of ~300µm. **Step 2 – Preparation of the glass slides.** Two glass slides (Microscope slides special size 76 x 52 mm, Marienfeld Superior) were used as substrates for the microfluidic chip. One of the slides was manually drilled (Dremel 4000 with Diamond grinding bit ⌀ 1.8mm, Proxxon) with multiple holes at locations for the inlets and outlets

of the interconnecting microchannels (according to the CAD design). This slide was then sonicated for 10min and washed several times in deionized (DI) water to remove any glass residue caused by the drilling. Then, both slides were cleaned in a Piranha solution (H_2SO_4(%):H_2O_2(%) = 3:1) for 10min, washed in DI water, and dried with a nitrogen gas blower. For purposes of the fabrication of polyelectrolyte-based diodes, the glass surfaces were functionalized with 3-(Trimethoxysilyl)propyl methacrylate (TMSMA, Sigma) to ensure a covalent link to the cured polyelectrolytes used in step 4[S1]. The functionalization was obtained by full immersion of the slides for 2 hours in a methanol solution containing 0.5% TMSMA and 0.5% acetic acid, followed by washing with a methanol solution and drying with a nitrogen gas blower. **Step 3- Assembling the microfluidic chip.** After peeling off one protective sheet of the adhesive and removing the adhesive tape that was located within the cut microfluidic channel areas, the rest of the adhesive was physically pressed against the drilled glass slide to bind them together while ensuring that the drilled holes overlapped with the inlet and outlet regions (ii). Then, the other protective sheet was peeled off from the adhesive, and the second glass slide was pressed so that the adhesive was sandwiched between the two slides (iii). **Step 4- fabrication of the bi-polar polyelectrolyte diode.** The procedure followed Han. J. et al. [S2], and Chun. H et al. [S3]. Two monomers were used to form oppositely charged polyelectrolytes that acted as a bipolar junction of a diode. For the positively and negatively charged polyelectrolytes, 4.2M Diallyldimethylammonium chloride (DADMAC, Sigma) and 5M 2-acrylamido- 2-methyl-1-propanesulfonic acid (AMPSA, Sigma) solutions containing 2% photoinitiator (2-Hydroxy-4′-(2-hydroxyethoxy)-2-methylpropiophenone, Sigma), and 2% cross linker(N,N′-Methylenebis(acrylamide), Sigma) were used. Initially, the entire microchannel was filled with a DADMAC (or AMPSA) solution (i). Then, the top slide was covered with a mask made of opaque tape impenetrable to light, where only half of the designated locations of the diode's junctions were left uncovered (ii). Next, the monomer solution was photopolymerized and cured by exposing the chip to UV light at an intensity of 10mW/cm^{-2} for 10s (or 20s) forming an immobilized gel of poly(DADMAC) (or poly(AMPSA)). The exposure times were optimized to gain the highest polymerization resolution, while ensuring homogeneous polymerization along the entire microchannel height. After exposure, the microchannels were washed several times with a 10mM KCl aqueous solution and cleaned with nitrogen gas to remove all the remaining non-polymerized DAMAC (or AMPSA) solution (iii). Finally, the same procedure was carried out with the AMPSA (or DADMAC) solution while the other side of the diode junctions were left uncovered (iv-vi). The microchip was stored wet in an aqueous 10mM KCl solution (vii).

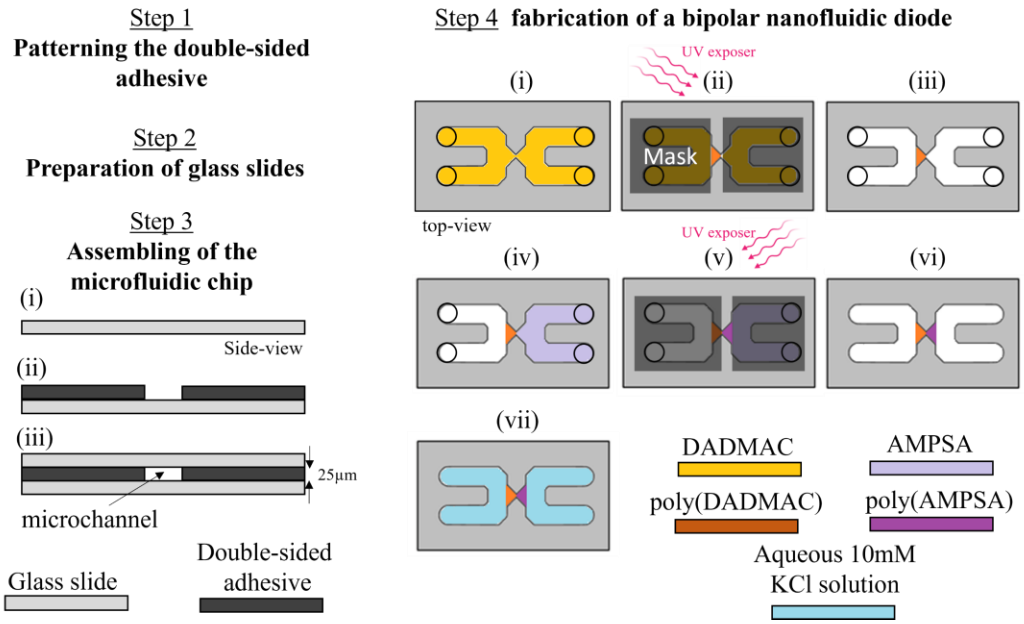

Figure S1: Fabrication procedure of the microfluidic chip and bipolar polyelectrolyte diode. The fabrication process consists of four main steps and allows for a controllable, rapid, and repeatable prototyping of a variety of microfluidic iontronic chips.

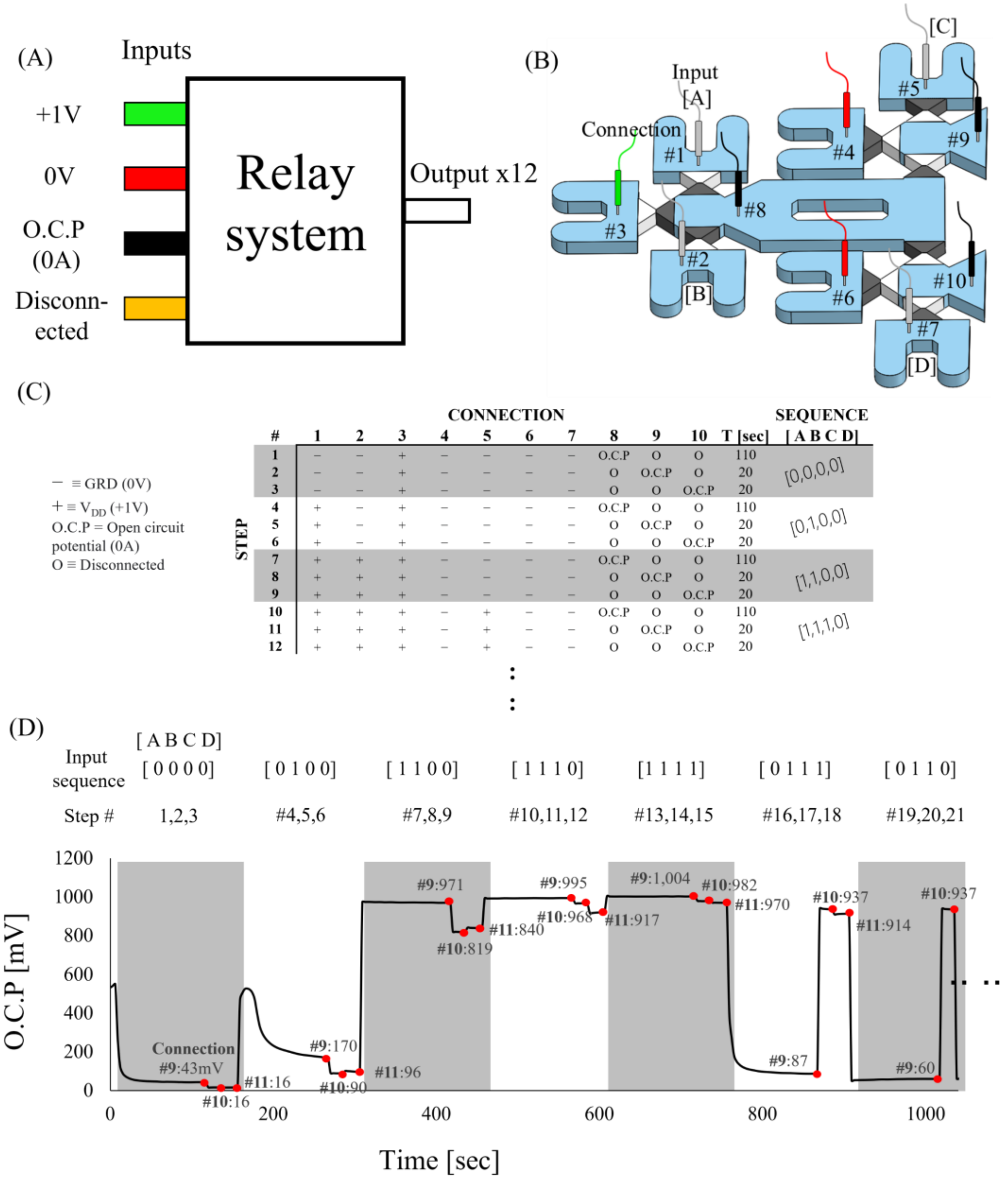

#	CONNECTION 1	2	3	4	5	6	7	8	9	10	T [sec]	SEQUENCE [A B C D]
1	−	−	+	−	−	−	−	O.C.P	O	O	110	[0,0,0,0]
2	−	−	+	−	−	−	−	O	O.C.P	O	20	
3	−	−	+	−	−	−	−	O	O	O.C.P	20	
4	+	−	+	−	−	−	−	O.C.P	O	O	110	[0,1,0,0]
5	+	−	+	−	−	−	−	O	O.C.P	O	20	
6	+	−	+	−	−	−	−	O	O	O.C.P	20	
7	+	+	+	−	−	−	−	O.C.P	O	O	110	[1,1,0,0]
8	+	+	+	−	−	−	−	O	O.C.P	O	20	
9	+	+	+	−	−	−	−	O	O	O.C.P	20	
10	+	+	+	−	+	−	−	O.C.P	O	O	110	[1,1,1,0]
11	+	+	+	−	+	−	−	O	O.C.P	O	20	
12	+	+	+	−	+	−	−	O	O	O.C.P	20	

Figure S2: Acquisition of the circuit's output readouts [Y]. (A) A custom-made relay system that automatically links four different inputs: V_{DD}=+1V, GRD=0V, 0A open circuit potential (O.C.P), and disconnected, to twelve individual outputs. **(B)** Schematic of one of the fluidic systems (AND-[OR||OR] circuit used in Fig.3). Each output is connected by a wire to an Ag/AgCl electrode situated at a specific location in the fluidic circuit. The connection numbers are indicated on the schematic. **(C)** The connection sequences are inserted into the relay system, where each row indicates the state of the connections (total of 10 connections in this circuit) and the operation time, T, in seconds. **(D)** The obtained O.C.P reading over time (black line), using a single Potentiostat (Gamry Reference 3000) and the relay system. The red dots indicate the steady-state readouts (in mV) of connections #9: [Y_1], #10: [Y_2], and #11: [Y_3] for each input sequence (inputs [A B C D]).

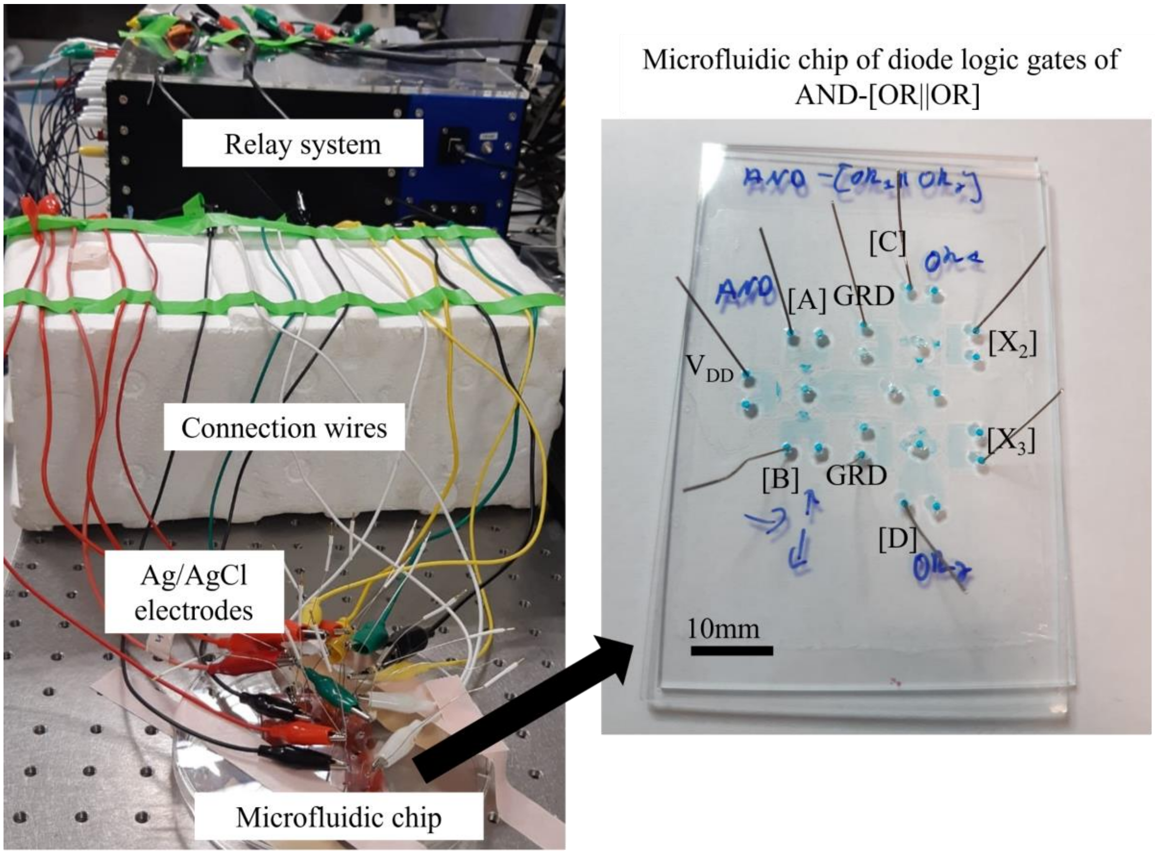

Figure S3: The experimental setup. Left: Photo of the entire measurement system, including the relay system, connection wires, Ag/AgCl electrodes, and the microfluidic chip. **Right:** Close-up of one of the microfluidic chips (in Fig.3) that contains a total of nine diodes, three diode-logic gates (AND-[OR||OR]), and ten microfluidic connections. The microchannels were filled with blue dye for visualization purposes.

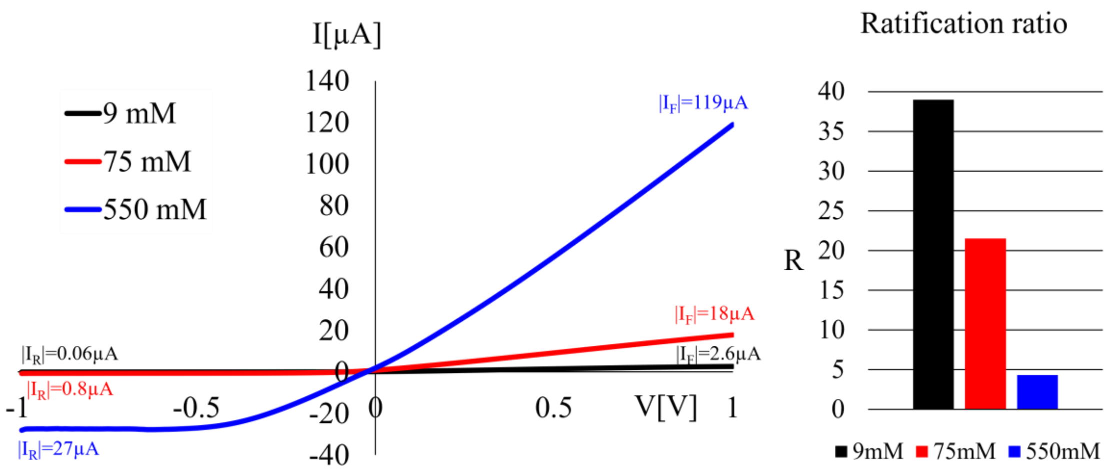

Figure S4: The effect of the electrolyte ionic strength on the bipolar diode performance. Left: Current-Voltage (I-V) response of the bipolar diode for three different ionic strengths (9, 75, 550mM marked in black, red, and blue, respectively). **Right:** The obtained rectification ratio, R, at ±1V.

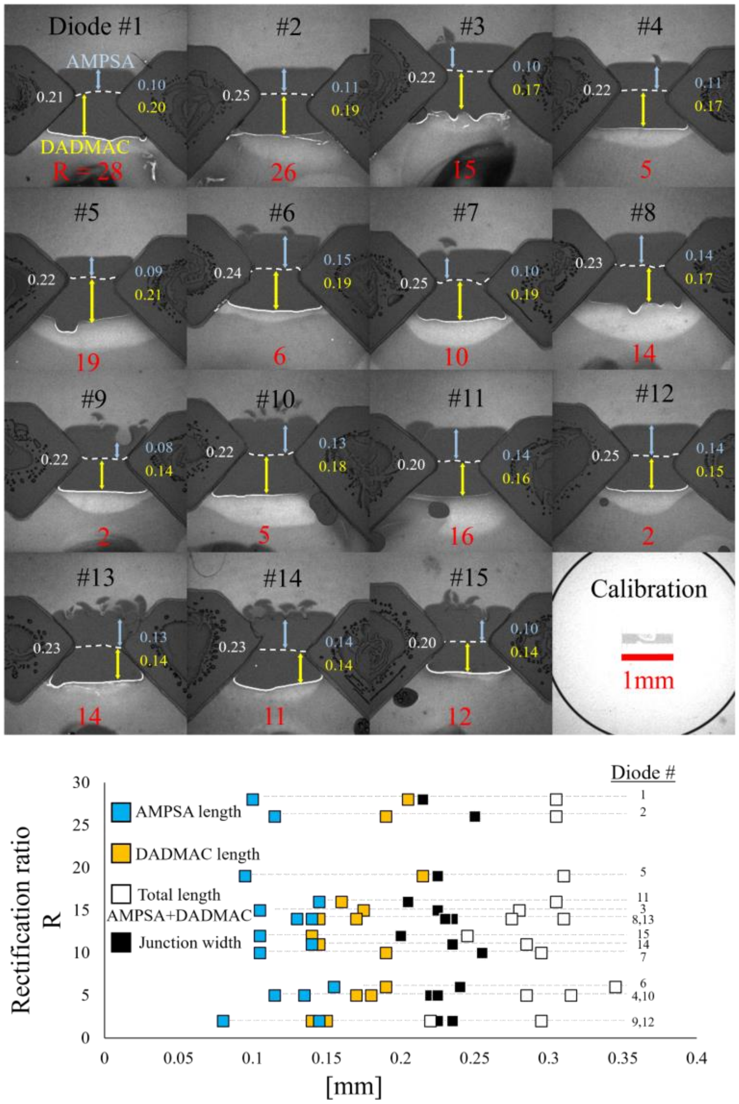

Figure S5: Variations in the bipolar polyelectrolyte diode fabrication and its influence on the rectification ratio. **Top:** Images taken under the microscope of 12 diodes (#1-12) showing the variations in fabrication results: the AMPSA membrane length (blue arrow), DADMAC membrane length (yellow arrow), the diode junction width (white line) and the corresponding rectification ratio, R, (in red). All dimensions are in mm. **Bottom:** The R obtained as a function of the variations in fabrication above. No clear trend was obtained, suggesting that there were defects and fluidic/ion transport bypasses that could not be visualized at this resolution.

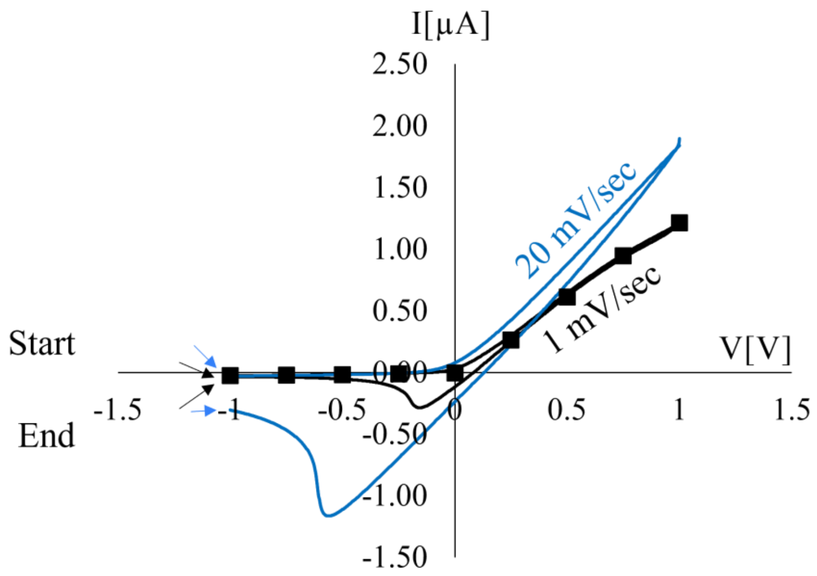

Figure S6: Current-Voltage (I-V) response of a single bipolar diode at two different scan rates: 20mV/sec (blue line), 1mV/sec (black line). Increasing the scanning rate resulted in increased hysteresis at the negative voltages when scanning from forward to reverse (+1V → –1V). The black rectangles show the steady state chronoamperometric responses obtained after 500sec (at V= –1, –0.75, –0.5, –0.25, 0, +0.25, +0.5, +0.75, +1V).

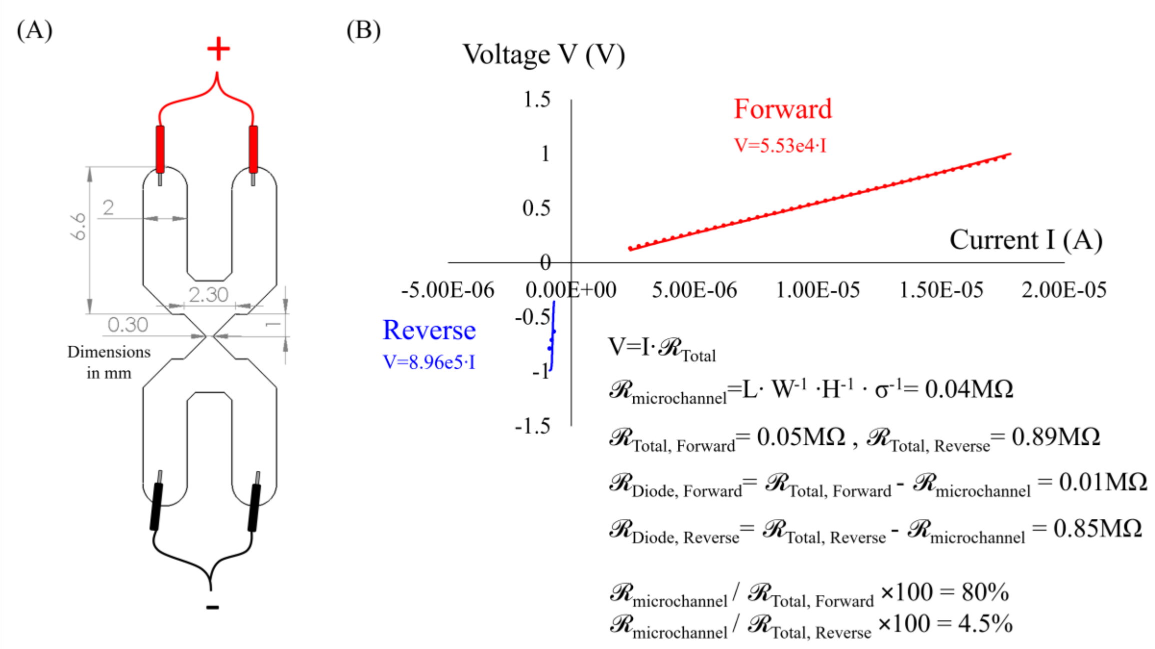

Figure S7: Estimation of the forward- and reverse-biased diode resistance contributions to the total measured resistance, $\mathscr{R}_{Total}$. (A) Schematic of a fluidic chip containing a single bipolar polyelectrolyte diode located between two interconnecting microchannels, where the electrodes were introduced at the ends of the microchannels. The channel dimensions are presented in mm. **(B)** Experimentally measured voltage vs. current response of the fluidic chip. The microchannel resistance, $\mathscr{R}_{Microchannel}$, was estimated based on the channel geometry (length L, width W, and height H) and the electrolyte's conductivity for 75mM KCl. The diode resistances, $\mathscr{R}_{Diode,Forward}$ and $\mathscr{R}_{Diode,Reverse}$, were calculated by subtracting the microchannel resistance from the overall measured resistance. Since the entire iontronic circuit shared the same electrolyte (i.e., the same conductivity), minimizing $\mathscr{R}_{Total}$ (without affecting the bipolar junction) was only possible through local geometry changes of the microchannel. This was achieved by shortening L and enlarging W, while keeping H uniform within the entire microfluidic chip so as not to complicate the fabrication process. In addition to $\mathscr{R}_{Total}$, the electrodes inserted into the microchannels' inlets may also have added undesired resistance due to their polarization or slow kinetics. To avoid these issues, we used Ag/AgCl electrodes with a large surface area and fast kinetics[S4].

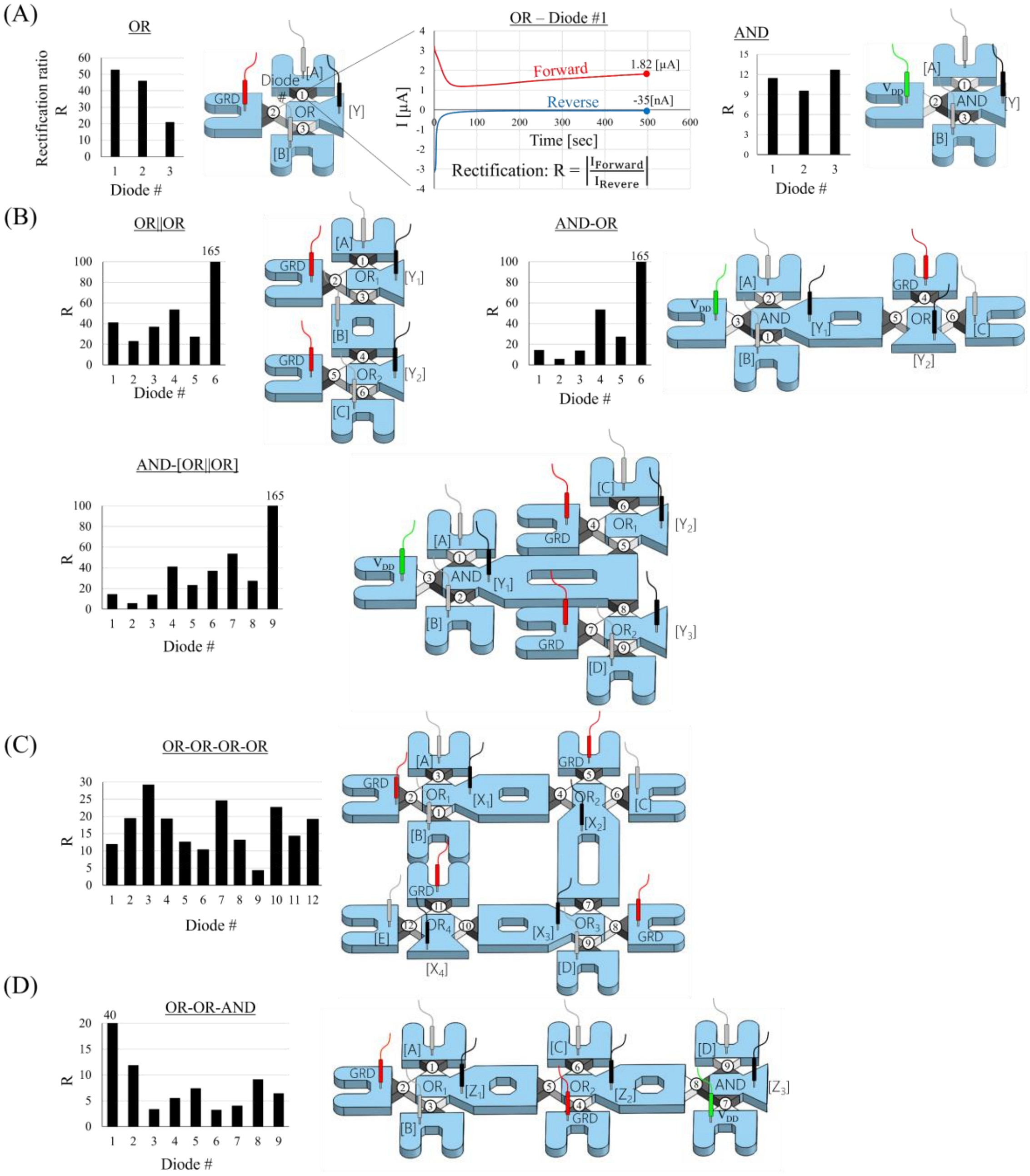

Figure S8: Rectification ratios, R, of the used bipolar polyelectrolyte diodes. **(A)** diodes used in Figure 2, **(B)** Figure 3, **(C)** Figure 4, **(D)** Figure S4. R is calculated as the ratio of the final current readouts ($|I_{Forward}/I_{Reverse}|$) of two chronoamperometric measurements at +1V and -1V ($I_{Forward}$ and $I_{Reverse}$, respectively) over 500sec. The diode numbers are indicated on the circuit schematics.

(A)

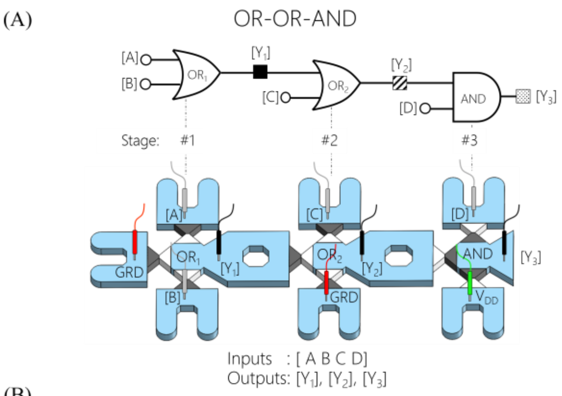

[A B C D]				[Y3] V	
[A]	[B]	[C]	[D]	Measured	Estimated
0	0	0	0	.20	.10
1	0	0	0	.21	.15
1	1	0	0	.20	.15
1	1	1	0	.21	.15
1	1	1	1	.92	.95
0	0	1	0	.20	.15
0	0	1	1	.87	.85
0	0	0	1	.47	.25
0	1	0	1	.61	.65
1	1	0	1	.66	.80
1	0	1	0	.20	.15

(B)

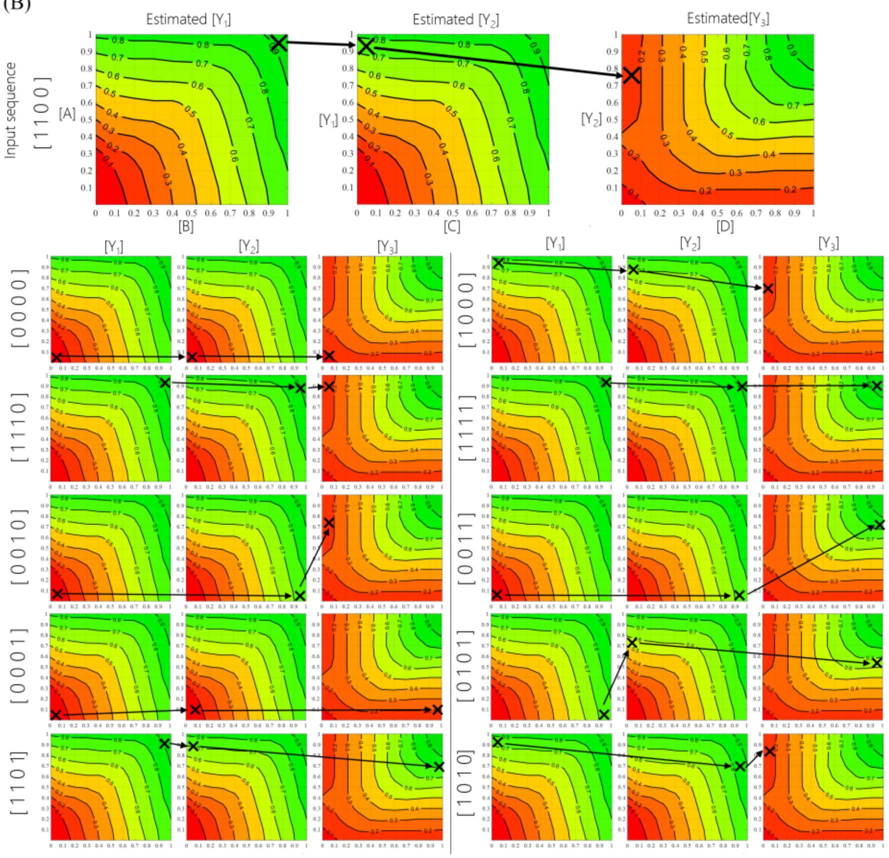

Figure S9: Successful integration of three diode-logic gates (DLGs) in series (OR-OR-AND) and prediction of circuit behavior. (A) Schematics of the gate, its fluidic implementation, and the resulting truth table showing correct logic interpretations of the 3rd gate's output readouts for all experimentally examined input sequences ([Y_3] 'Measured' column). **(B)** Prediction of the circuit behavior based on the individual DLG responses (response diagrams taken from Fig.2), where each diagram represents a DLG. The same input sequences were examined on the response diagrams, and exhibited a similar trend as the experimental results, with an average deviation of 75mV between the predicted outputs ([Y_3] 'Predicted' column in the truth table) and the measured outputs. X marks the predicted output voltages, and the arrows indicate the signal propagation path.

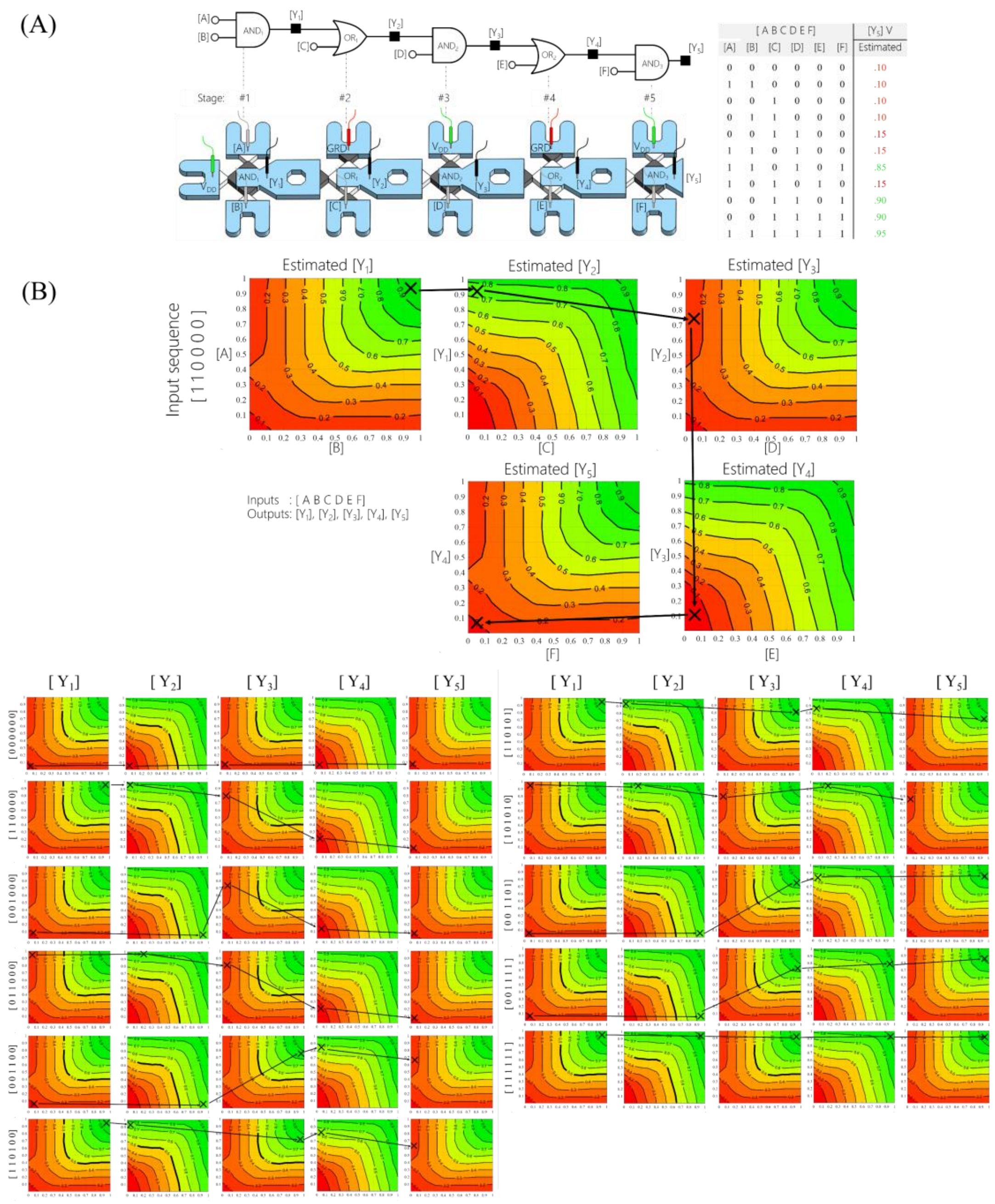

[A]	[B]	[C]	[D]	[E]	[F]	[Y5] V Estimated
0	0	0	0	0	0	.10
1	1	0	0	0	0	.10
0	0	1	0	0	0	.10
0	1	1	0	0	0	.10
0	0	1	1	0	0	.15
1	1	0	1	0	0	.15
1	1	0	1	0	1	.85
1	0	1	0	1	0	.15
0	0	1	1	0	1	.90
0	0	1	1	1	1	.90
1	1	1	1	1	1	.95

Figure S10: Theoretical output prediction of the integration of five diode-logic gates (DLGs) connected in series (AND-OR-AND-OR-AND). (A) Schematics of the gate, its potential fluidic implementation, and the resulting truth table showing correct logic interpretations of the 5th gate's estimated output readouts for all input sequences examined in (B). **(B) Theoretical** prediction of the circuit behavior based on the individual DLG responses (response diagrams taken from Fig.2). The input sequences were examined on the response diagrams, where the diagram represents a DLG. X indicates the predicted output voltages, and the arrows indicate the signal propagation path.

1N5711 Specifications

Characteristics		Typical Value	Unit	Note
Rectification factor,	R	$>10^5$		@ Absolute voltage of 1V
Reverse current,	I_R	200	µA	@ Voltage of -50V
Reverse breakdown voltage,	V_{BR}	-70	V	@ I_R=10 µA
Transition voltage,	V_{TR}	0.4	V	@ I_F=1mA
Maximum forward voltage,	V_{MAX}	70	V	
RC time constant,	τ_{RC}	1	ns	

Table S1: Typical values of solid-state p-n electronic diode type 1N5711. Taken from electronics-notes.com [S5]

[A]	[B]	[C]	[D]	[E]	[Y_1]	[Y_2]	[Y_3]	[Y_4]
0	0	0	0	0	.00	.00	.00	.00
1	0	0	0	0	.78	.51	.30	.25
1	1	0	0	0	.88	.60	.38	.33
0	0	1	0	0	.05	.79	.53	.47
0	0	0	1	0	.00	.00	.87	.79
0	0	0	0	1	.00	.00	.00	.89
0	1	0	0	1	.75	.54	.39	.91
0	0	1	0	1	.00	.81	.59	.93
0	0	0	1	1	.00	.00	.91	.94
0	0	1	1	1	.00	.91	.94	.94
0	1	1	1	1	.87	.93	.94	.94
1	1	1	1	1	.97	.95	.95	.94
0	0	1	0	0	.01	.81	.55	.48
0	0	1	1	0	.00	.91	.91	.83
1	1	0	1	0	.93	.79	.90	.82
1	0	0	0	1	.81	.58	.43	.92
1	0	0	1	0	.87	.75	.90	.82
0	0	1	1	0	.11	.91	.91	.83
0	1	1	1	0	.88	.93	.92	.83
0	1	0	0	1	.76	.55	.40	.92
1	1	0	0	1	.89	.95	.48	.92

Table S2: Truth table for DLG integration in Fig.4 (OR-OR-OR-OR). The voltage readouts ([Y_1], [Y_2], [Y_3], [Y_4]) are in Volt. Red and green represent correct interpretations of low and high logic levels, respectively, and yellow corresponds to a faulty logic interpretation.

References

[S1] Garoff, Henrik, and Wilhelm Ansorge. "Improvements of DNA sequencing gels." *Analytical biochemistry* 115.2 (1981): 450-457.

[S2] Han, Ji-Hyung, et al. "Ionic circuits based on polyelectrolyte diodes on a microchip." *Angewandte Chemie International Edition* 48.21 (2009): 3830-3833.

[S3] Chun, Honggu, Taek Dong Chung, and Hee Chan Kim. "Cytometry and velocimetry on a microfluidic chip using polyelectrolytic salt bridges." Analytical chemistry 77.8 (2005): 2490-2495.

[S4] Dunphy-Guzmán, K. A.; Karnik, R. N.; Newman, J. S.; Majumdar, A. Spatially Controlled Microfluidics Using Low-Voltage Electrokinetics. *J. Microelectromechanical Syst.* **2006**, *15* (1), 237–245. https://doi.org/10.1109/JMEMS.2005.863789.

[S5] https://www.electronics-notes.com/articles/electronic_components/diode/specifications-parameters-ratings.php#:~:text=The%20leakage%20current%20characteristic%20or,low%20before%20reverse%20breakdown%20occurs .
